# The role of the pulmonary function laboratory in the assessment of adults with neuromuscular disease

**DOI:** 10.36416/1806-3756/e20250417

**Published:** 2025-11-19

**Authors:** Danilo C Berton, Denis E O’Donnell, José Alberto Neder

**Affiliations:** 1. Unidade de Fisiologia Pulmonar, Hospital de Clínicas de Porto Alegre, Universidade Federal do Rio Grande do Sul, Porto Alegre (RS) Brasil.; 2. Pulmonary Function Laboratory and Respiratory Investigation Unit, Division of Respirology, Kingston Health Science Center & Queen’s University, Kingston (ON) Canada.

## BACKGROUND

**Figure 1:**
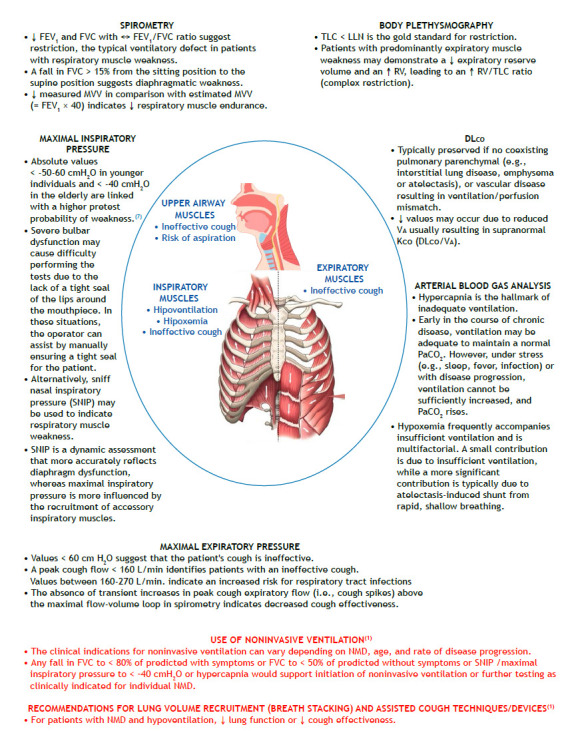
Involvement of inspiratory, expiratory, and/or upper airway muscles in patients with neuromuscular disease (NMD) determines the predominating clinical presentation (in blue). Different pulmonary function tests (in black) can reveal functional impairments and support the indication of specific therapies (in red). MVV: maximal voluntary ventilation; LLN: lower limit of normal; ¯: decreased; ­: increased; «: preserved; V_A_: alveolar volume; and K_CO_: carbon monoxide transfer coefficient.

Neuromuscular disease (NMD) can affect all respiratory muscle groups, and respiratory complications are the major cause of morbidity and mortality.^1^ The duration of symptoms varies depending on the underlying diagnosis. NMD can be acute (e.g., Guillain-Barré syndrome; acute spinal cord or phrenic nerve trauma or infarction; epidural abscess; acute poisoning; drug-related NMD; metabolic disturbances; tetanus or other infections; and acute myasthenic crisis) or present slowly over months (e.g., amyotrophic lateral sclerosis, multiple sclerosis, spinal cord tumors, myasthenia gravis, syringomyelia, muscular dystrophy, and myotonic dystrophy). In the latter context, pulmonary function tests (PFTs) play a prominent role in objectively assessing respiratory muscle strength and potential consequences of weakness of the respiratory system (Figure 1). 

## OVERVIEW

A 55-year-old overweight man (BMI = 27 kg/m^2^) with a history of heavy smoking (30 pack-years) was referred for a pulmonology consultation because of long-standing sporadic inclusion body myositis. He reported having experienced leg pain and weakness since he was in his 30s and 40s, respectively. Although there were no respiratory symptoms during wakefulness (with a modified Medical Research Council scale score of 1, with no cough or phlegm) or daytime somnolence (an Epworth Sleepiness Scale score of 6), the patient did report episodes of nocturnal choking and frequent rhonchi. Moderate left convex scoliosis was observed on physical examination. PFTs indicated a restrictive ventilatory defect (an FVC of 62% of the predicted value and a TLC of 68% of the predicted value) and respiratory muscle weakness (an MIP of 57% of the predicted value and an MEP of 69% of the predicted value). Of note, RV and the RV/TLC ratio were within and above the upper limit of normal, respectively. A proportional reduction in DL_CO_ (60% of the predicted value) and alveolar volume (V_A_; 65% of the predicted value) corresponded to a carbon monoxide transfer coefficient (K_CO_) within normal ranges (94% of the predicted value = a z-score of −0.30). Diffuse myocardial hypokinesis (a left ventricular ejection fraction of 49%) was observed on echocardiography. Mild obstructive respiratory disorder was observed during overnight polysomnography (an apnea-hypopnea index of 14.6 events/h), with significant CO_2_ retention (mean partial pressure of end-tidal CO_2_ = 39, with peaks of 51 mmHg). 

Restriction is the typical finding in patients with respiratory muscle weakness. It is suggested by reduced FEV_1_ and FVC with a preserved FEV_1_/FVC ratio and confirmed by a reduced TLC. In cases of preserved FVC, a fall > 15% in FVC from the sitting position to the supine position supports a diagnosis of diaphragm weakness.^2^ This threshold can be higher in the presence of concomitant ventilatory defects.^3^ A high RV/TLC ratio was a consequence of a low TLC (rather than a high RV), in keeping with restriction. Nevertheless, when the expiratory muscles are involved, RV and RV/TLC may be increased, resulting in complex restriction (reduced FVC relative to TLC).^4^ DL_CO_ is reduced in extraparenchymal restriction as a result of reduced V_A_, which would lead to a supranormal K_CO_ (DL_CO_/V_A_). A “normal” K_CO_ with preserved V_A_/TLC indicates some degree of concomitant intraparenchymal restriction.^5^ In the current case, it was attributed to alveolar fibrosing sequelae from repeated episodes of pulmonary congestion caused by cardiomyopathy. Arterial blood gas analysis should be routinely obtained to determine whether daytime hypercapnia is present. Hypercapnia, however, may be evident during sleep only, when polysomnography with end-tidal or transcutaneous capnography is useful. 

## CLINICAL MESSAGE

PFTs are regularly recommended for patients with NMD who may exhibit varying rates of decline in lung function.^1^ Objective testing is important because there is no correlation between respiratory muscle weakness and the degree of peripheral muscle weakness in several conditions.^6^ Functional testing helps identify patients who need specific therapies, such as assisted cough, airway clearance, and ventilatory support.^1^
